# Differential Role of Autophagy in CD4 T Cells and Macrophages during X4 and R5 HIV-1 Infection

**DOI:** 10.1371/journal.pone.0005787

**Published:** 2009-06-03

**Authors:** Lucile Espert, Mihayl Varbanov, Véronique Robert-Hebmann, Sophie Sagnier, Ian Robbins, Françoise Sanchez, Virginie Lafont, Martine Biard-Piechaczyk

**Affiliations:** 1 CPBS, UM1, UM2, CNRS, Institut de Biologie, 4, CS 69033, Montpellier, France; 2 Institut de génétique Moléculaire, Montpellier, France; Institut Pasteur Korea, Republic of Korea

## Abstract

**Background:**

HIV-1 can infect and replicate in both CD4 T cells and macrophages. In these cell types, HIV-1 entry is mediated by the binding of envelope glycoproteins (gp120 and gp41, Env) to the receptor CD4 and a coreceptor, principally CCR5 or CXCR4, depending on the viral strain (R5 or X4, respectively). Uninfected CD4 T cells undergo X4 Env-mediated autophagy, leading to their apoptosis, a mechanism now recognized as central to immunodeficiency.

**Methodology/Principal Findings:**

We demonstrate here that autophagy and cell death are also induced in the uninfected CD4 T cells by HIV-1 R5 Env, while autophagy is inhibited in productively X4 or R5-infected CD4 T cells. In contrast, uninfected macrophages, a preserved cell population during HIV-1 infection, do not undergo X4 or R5 Env-mediated autophagy. Autophagosomes, however, are present in macrophages exposed to infectious HIV-1 particles, independently of coreceptor use. Interestingly, we observed two populations of autophagic cells: one highly autophagic and the other weakly autophagic. Surprisingly, viruses could be detected in the weakly autophagic cells but not in the highly autophagic cells. In addition, we show that the triggering of autophagy in macrophages is necessary for viral replication but addition of Bafilomycin A1, which blocks the final stages of autophagy, strongly increases productive infection.

**Conclusions/Significance:**

Taken together, our data suggest that autophagy plays a complex, but essential, role in HIV pathology by regulating both viral replication and the fate of the target cells.

## Introduction

HIV-1 can infect and replicate in both CD4 T cells and macrophages, however, only CD4 T lymphocytes are depleted during HIV-1 infection. This indicates that several aspects of the virus-host relationship are different between CD4 T cells and macrophages [1]. Whatever the cell type, HIV-1 entry is mediated by the binding of envelope glycoproteins (gp120 and gp41, Env) to the receptor CD4 and a coreceptor, principally CCR5 or CXCR4. gp120 interacts initially with CD4 and then with the coreceptor. This leads to gp41 insertion into the target membrane and membrane fusion. Coreceptor use is correlated, at least in part, with the different phases of the disease. R5 strains, which use CCR5 for entry, are responsible for the primary infection while X4 strains, which use CXCR4, emerge later in the course of infection and are associated with a severe depletion of CD4 T cells [2]. Infection by both cell-free viruses and by cell-to-cell contact between infected and uninfected cells, involving the formation of virological synapses, leads to the spread of HIV. Viral transfer, however, appears to be more efficient after cell-to-cell interaction [3], [4], [5].

During cell-to-cell contact, the binding of X4 Env, expressed on the infected cells, to CXCR4, expressed on the uninfected CD4 T cells, is known to trigger apoptosis of the latter [6], [7], [8]. This mechanism is most likely responsible for immunodeficiency. We have previously demonstrated that, independently of HIV-1 replication, Env-transfected or HIV-1-infected cells, both of which express X4 Env at the cell surface, induce autophagy in uninfected CD4 T lymphocytes *via* the fusogenic function of gp41 [9]. This induction of autophagy is prerequisite to Env-induced apoptosis [10].

Autophagy is a membrane trafficking process involved in cellular homeostasis through protein degradation and organelle turnover. This process is characterized by the formation of double membrane vacuoles, named autophagosomes, which engulf cytoplasmic material. Following maturation, autophagosomes fuse with lysosomes to digest the sequestered material [11]. Autophagy is also recognised as a mechanism involved in innate and adaptative immunity against pathogens [12]. In this context, it has been proposed as a protective mechanism against infections through the degradation of pathogens in autolysosomes [13], [14]. Certain pathogens, however, can exploit autophagy to replicate more efficiently [15], [16].

Despite the efforts made to understand how HIV-1 selectively kills CD4 T cells, leaving intact the population of macrophages, many questions remain unanswered. In addition, some controversy still exists about the cytopathic effects of X4 and R5 HIV-1 strains. The emergence of X4 strains in HIV-1-infected patients correlates with a rapid progression to AIDS. Differences in the distribution of CCR5 and CXCR4 on the CD4 T cells, however, may also explain these results. Indeed, CCR5 is expressed on 5 to 25% of peripheral blood CD4 T cells while CXCR4 is expressed on nearly all peripheral blood CD4 T cells [17], [18], [19], [20].

We have already demonstrated that autophagy is an important mechanism involved in the demise of uninfected bystander CD4 T cells, via CXCR4. It remains to be established, however, whether the same is true when the CCR5 coreceptor is used. In addition, macrophages, that express both CXCR4 and CCR5, are also infected by HIV-1, but are not depleted.

We, therefore, analyzed autophagy in CD4 T cells and macrophages, the two main target cells of HIV-1, during infection with either X4 or R5 strains. We first established that Env triggers autophagy and cell death in uninfected CD4 T cells, whatever the coreceptor use. In contrast, uninfected macrophages, a cell population known to be resistant to cell depletion in patients, do not undergo X4 or R5 Env-mediated autophagy and cell death. Furthermore, during HIV-1 productive infection, we demonstrate that autophagy is repressed in CD4 T cells but induced and regulated in macrophages. Autophagy is thus differentially used and controlled in HIV-1-infected cells, and consequently may govern the course of the disease.

## Results

### Cellular coculture models to analyze Env-mediated autophagy of uninfected cells

**Figure 1:**
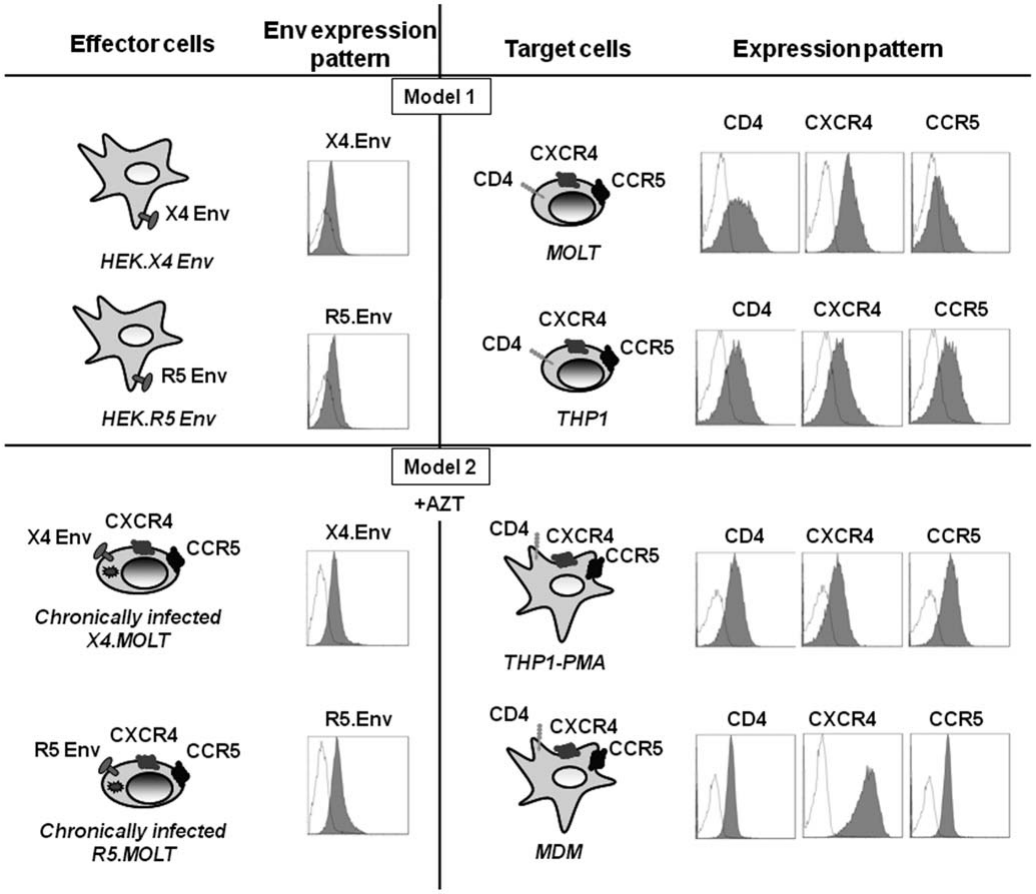
Description of the cellular models. In model 1, target CD4 T cells (MOLT-4) or the monocytic cell line THP1 are cocultured with effector HEK cells expressing, or not, X4 or R5 Env. In model 2, THP1-PMA or MDM are cocultured with chronically X4 or R5-infected MOLT-4 or parental MOLT-4 in the presence of AZT to block viral replication. Expression of gp120 at the surface of HEK.X4 Env, HEK.R5 Env, and chronically X4 and R5 infected MOLT-4 (grey histograms) as well as parental HEK and MOLT-4 cells, used as negative controls (white histograms), were analyzed by flow cytometry after incubation of the cells with PBS containing anti-gp120 human polyclonal Ab. Bound Ab was detected using a secondary FITC-labeled goat anti-human Ig. Expression analysis of the extracellular domains of CD4, CXCR4 and CCR5 at the surface of target cells was performed after incubation of the cells with PBS (white histograms) or with anti-CD4, anti-CXCR4 or anti-CCR5 (grey histograms) mAb at 10 µg/mL. Bound mAb was detected using FITC-labeled goat anti-mouse Ig. The fluorescence intensity was recorded in the log mode on an EPICS XL4 cytofluorometer.

We have used cellular models that involve cell-to-cell contact, as this is known to be important in HIV-1 pathogenesis. Two models (Figure 1) were used to analyze the capacity of X4 and R5 Env expressed on cells to trigger autophagy in the absence of viral replication. They are based on the coculture of effector cells, which express Env, with target cells that express CD4, CXCR4 and CCR5. These models were created to facilitate the analysis of Env-induced autophagy in both CD4 T cells and macrophages since target and effector cells can be easily separated as one is adherent with the other in suspension.

In model 1, the target cells (in which autophagy was analyzed) were either CD4 T cells or the human monocytic leukemia THP1 cell line. As expression of CCR5 is highly variable on primary CD4 T cells [21], we used MOLT-4 cells, a human CD4 T cell line that homogeneously expresses CXCR4 and CCR5. These target cells were cocultured with effector HEK.293 cells that stably express X4 or R5 Env (HEK.X4 Env or HEK.R5 Env, respectively). This model has previously been used to analyze X4 Env-mediated autophagy in CD4 T cells [10]. To analyze the capacity of R5 Env to trigger autophagy in target cells, we constructed stable HEK.293 cells that express R5 Env at the same level as HEK.X4 Env. As a negative control, we used the parental HEK cell line or a stable HEK line containing the parental vector backbone.

In model 2, the target cells were PMA-differentiated, THP1 cells (THP1-PMA) and primary, monocyte-differentiated, macrophages (MDM). The effector cells were chronically HIV-1-infected MOLT-4 cells (X4 and R5 strains). Uninfected MOLT-4 cells were used as a negative control. Azidothymidine (AZT; 1 µM), an inhibitor of reverse transcriptase, was added to the coculture to inhibit viral replication. Expression patterns of Env, on the effector cells, and CD4, CXCR4 and CCR5, on the target cells, are shown in Figure 1.

### R5 Env expressed on effector cells induces autophagy and cell death of uninfected CD4 T cells

**Figure 2:**
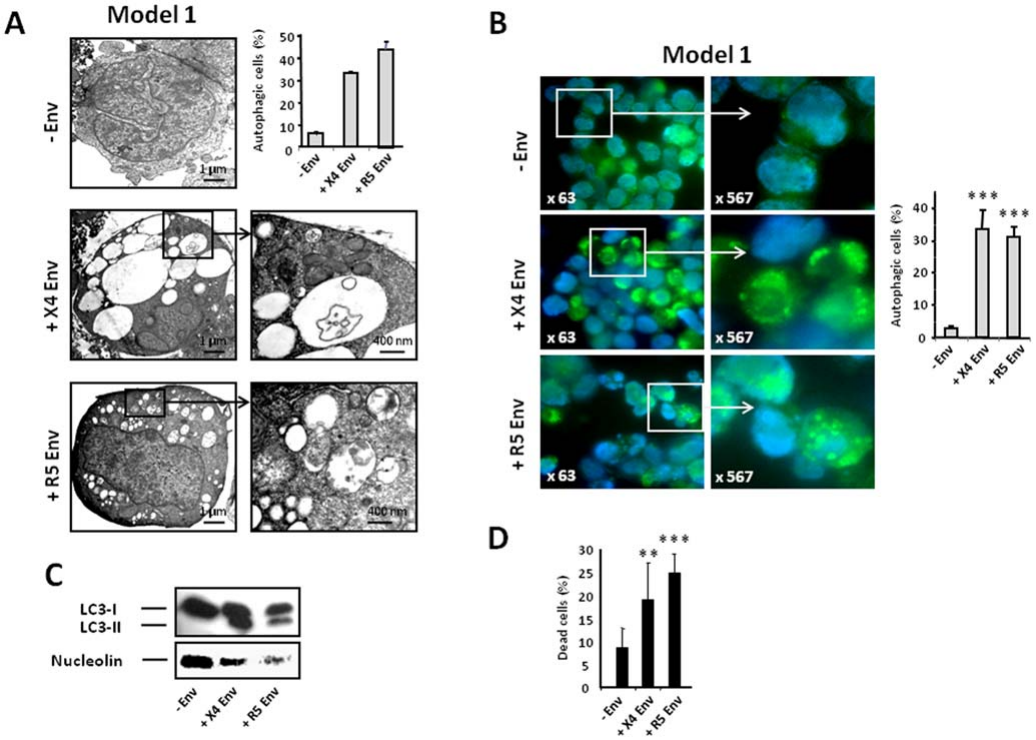
X4 and R5 Env expressed on cells induce autophagy and cell death of uninfected CD4 T cells. (A) MOLT-4 cells were cocultured for 3 days with HEK, HEK.Env X4 or HEK.Env R5 and examined by TEM. The percentage of autophagic cells in the target cell sections was analyzed from at least 100 randomly chosen TEM fields by 2 investigators (indicated enlargements). Data are representative of at least 2 independent experiments. (B) MOLT-4 cells were transiently transfected with GFP-LC3, cocultured for 2 days with HEK, HEK.Env X4 or HEK.Env R5 MOLT-4 and examined by epifluorescence. Data are representative of 3 independent experiments; more than 100 cells were counted by 2 investigators. Cells with autophagosomes were defined as cells that had 5 or more LC3 spots in the cytoplasm. Magnification is shown. (C) Immunoblot analysis of LC3-I to LC3-II conversion in MOLT-4 cells was performed after coculture for 2 days with HEK, HEK.Env X4 or HEK.Env R5. (D) Global cell death of MOLT-4 cells cocultured for 3 days with HEK, HEK.Env X4 or HEK.Env R5 was determined using the trypan blue exclusion test. Data are representative of at least 5 independent experiments. **P<0.01; ***P<0.001.

We first analyzed the capacity of R5 Env to trigger autophagy in MOLT-4 cells using model 1 (Figure 1). Autophagy was triggered in these uninfected CD4 T cells cocultured with effector cells expressing either X4 Env or R5 Env, as visualized by transmission electron microscopy (TEM) (Figure 2A), fluorescence studies (Figure 2B), and western blot analysis of LC3 -II expression (Figure 2C), indicating that R5 Env, like X4 Env, is able to trigger autophagy in the uninfected CD4 T cells. In addition, like X4 Env, R5 Env triggered CD4 T-cell death (Figure 2D). These results were confirmed by using another CD4 T cell line that stably expresses CXCR4 and CCR5 (A2.01/CD4/CCR5). These cells also underwent X4- and R5-mediated autophagy and cell death (data not shown).

These results corroborate and expand our previous results demonstrating that the fusogenic function of gp41 is responsible for Env-mediated autophagy and that the hemifusion process is sufficient to trigger autophagy and cell death [22]. Indeed, the interaction of gp120 with CD4 and the coreceptor, irrespective of the coreceptor used, induces a structural rearrangement in gp41 and its insertion into the target membrane.

### X4 and R5 Env do not trigger autophagy and cell death in uninfected cells from the monocyte/macrophage lineage

**Figure 3:**
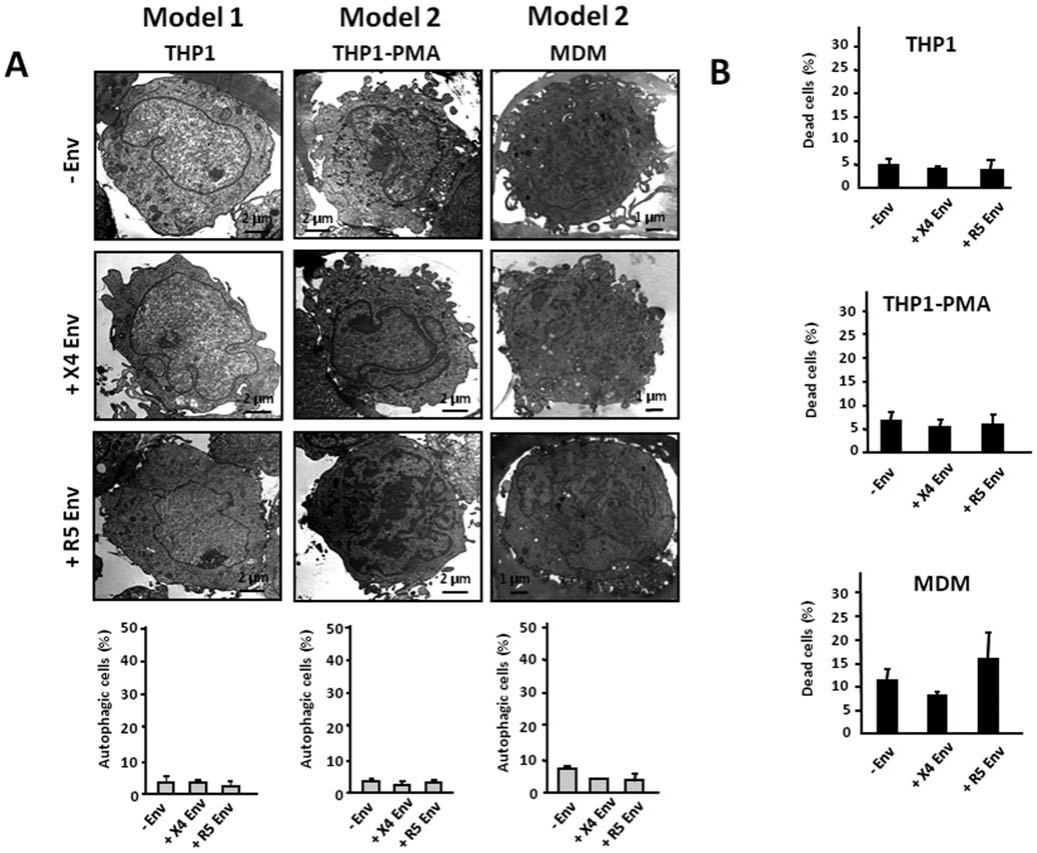
R5 and X4 Env expressed on cells do not trigger autophagy and cell death in cells from the monocyte/macrophage lineage. (A) THP1, and THP1-PMA and MDM were cocultured for 3 days with effector cells that express, or not, X4 or R5 Env using the models 1 and 2, respectively. Target cells were then examined by TEM as described in Figure 1. (B) Global cell death of THP1, and THP1-PMA and MDM cocultured for 3 days with effector cells that express, or not, X4 or R5 Env was determined using the trypan blue exclusion test. Data are representative of at least 5 independent experiments.

In addition to CD4 T cells, cells from the monocyte/macrophage lineage are also major targets of HIV-1 infection. X4 and R5-mediated autophagy was thus analyzed by TEM in THP1 using model 1, and in both THP1-PMA and MDM using model 2. Surprisingly, X4 and R5 Env did not trigger autophagy of THP1, THP1-PMA and MDM (Figure 3A), whereas these target cells were still susceptible to autophagy induced by rapamycin (Rapa), a classical inducer of autophagy (data not shown). Furthermore, X4 or R5 Env expressed on effector cells did not induce the death of THP1, THP1-PMA and MDM (Figure 3B). Similarly, the human monocytic cell line U937 did not undergo Env-induced autophagy and cell death (data not shown).

### Autophagy is repressed in CD4 T cells that are productively infected by both X4 and R5 HIV

**Figure 4:**
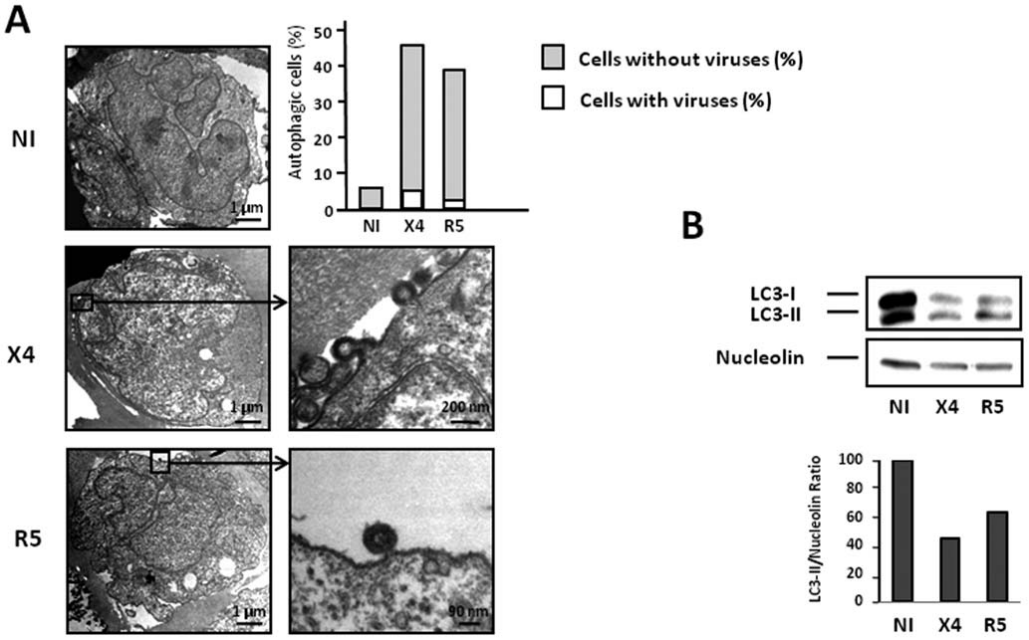
HIV-1 X4 and R5-infected CD4 T cells do not undergo autophagy. (A) MOLT-4 cells were cocultured with HEK producing, or not, infectious X4 and R5 viruses for 3 days and examined by TEM as described in Figure 1. 56.7% and 47.1% of MOLT-4 cells were infected by X4 and R5 HIV-1, respectively. The percentage of autophagic cells presenting, or not, budding virions after X4 or R5 exposure are shown. HIV-1 productively infected MOLT-4 cells are presented, with a close up of budding viruses on the right-hand side. (B) Immunoblot analysis of LC3-I to LC3-II conversion in MOLT-4 cells was performed after infection for 1 day with free X4 and R5 viruses. The LC3-II/Nucleolin ratio was calculated as the intensity of the LC3-II immunoblot compared with the intensity of the Nucleolin immunoblot using ImageJ. Data are representative of at least 3 experiments.

Although CXCR4 is expressed by the large majority of primary CD4-T cells, only 5–25% express CCR5. In addition, the level of expression of CCR5 is highly variable from one individual to another. We therefore preferred to analyze autophagy in productively infected MOLT-4 CD4 T cells, which express both coreceptors. Autophagy is triggered in these cells when they are cocultured with Env-expressing HEK.293 effector cells (Figure 2). When MOLT-4 cells were cocultured with the HEK.293 cells transfected with X4 NL4-3 or R5 NL4-Ad8 DNA constructs that produce infectious virions, two target cell populations were observed: a population of highly autophagic cells, that did not present detectable budding virions at the cell surface by TEM, and a non autophagic population that was productively infected (cells with budding virions) (Figure 4A). The percentages of highly autophagic cells correlate with those obtained in the uninfected CD4 T cells cocultured with Env-expressing cells (Figure 2A, model 1). Furthermore, the level of LC3-II decreased in the MOLT-4 cells after infection by either X4 or R5 strains, suggesting that HIV-1 infection actively inhibits the Env-mediated autophagic process (Figure 4B). Interestingly, the level of LC3-I also decreased in MOLT-4 cells after infection with either X4- or R5 strains, suggesting a role for HIV-1 in regulating its expression. Thus exposure of CD4 T cells to infectious X4 and R5 viruses blocks autophagy that is induced during cell-to-cell contacts between Env expressed on infected cells and its receptors, CD4 and the coreceptor, expressed on target cells.

### Autophagy is induced in the monocyte/macrophage cell lineage after X4 or R5 infection

**Figure 5:**
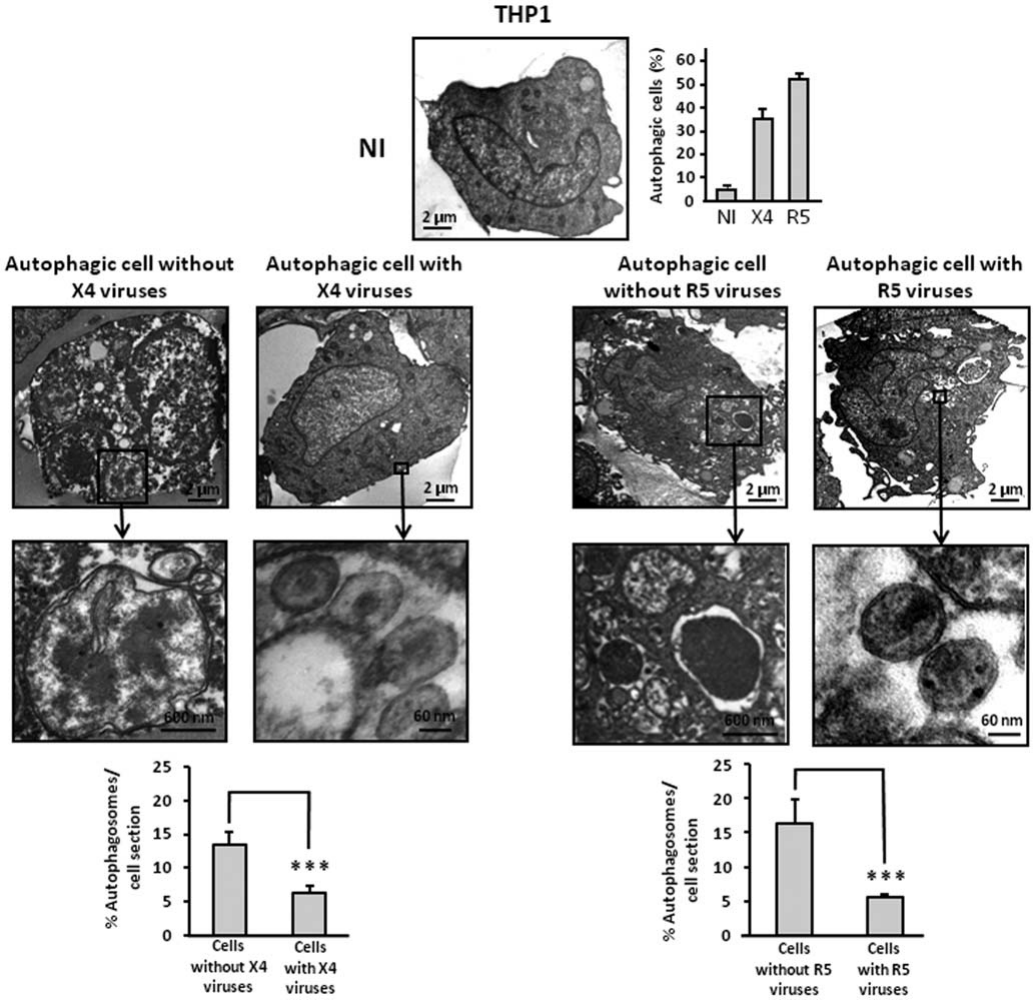
X4 and R5-infected THP1 undergo autophagy. THP1 were cocultured for 3 days with parental HEK cells (NI = not infected), or with HEK cells transfected with X4 NL4-3 or R5 NL4-Ad8 DNA constructs that produce infectious virions. Cells were examined by TEM by 2 independent investigators. At least two independent experiments were done. The upper graph indicates the percentage of autophagic cells. The presence of intact virions and the number of autophagosomes in the target cell sections were also analyzed. Enlargements of viruses and autophagosomes from cells exposed to viruses are shown.

**Figure 6:**
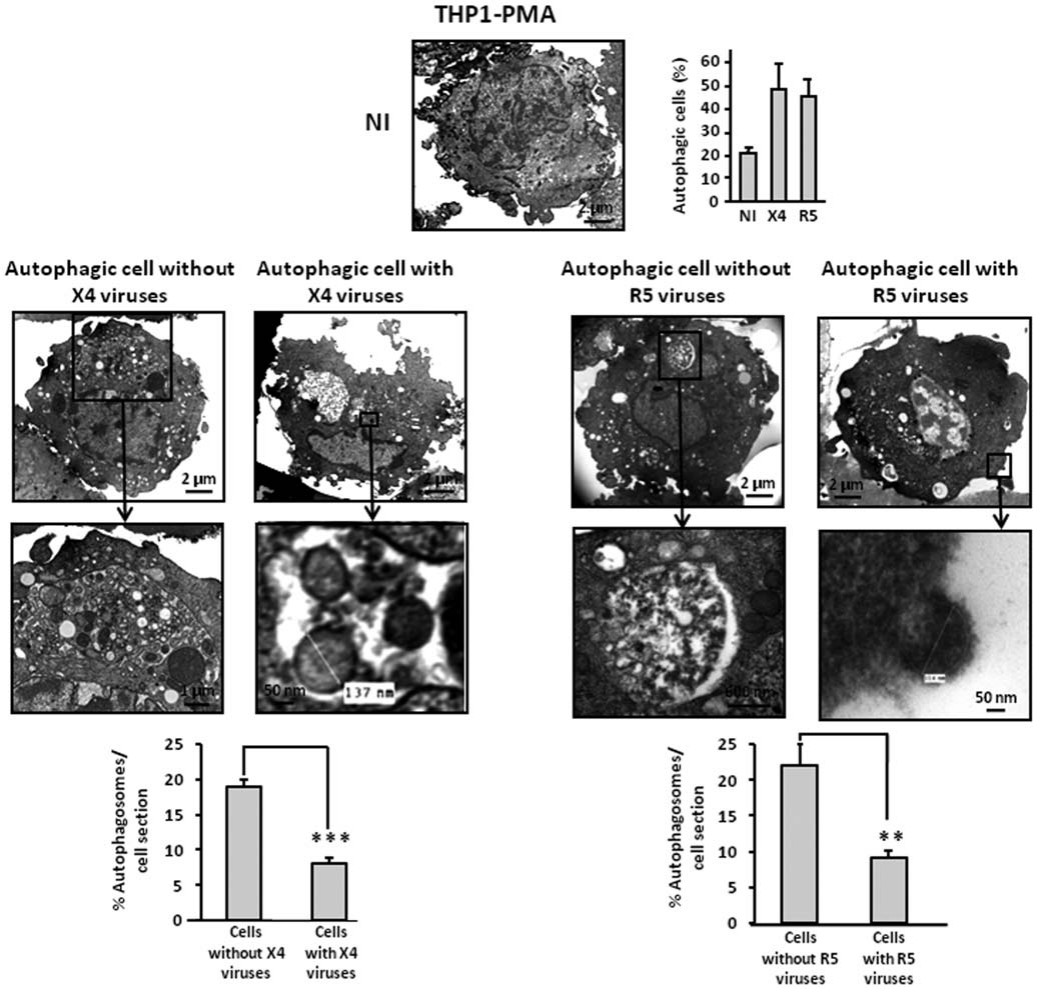
X4 and R5-infected THP1-PMA undergo autophagy. THP1-PMA were cocultured for 3 days with chronically X4 or R5-infected MOLT-4, or with uninfected MOLT-4, and examined by TEM as described in Figure 5. Enlargement of viruses and autophagosomes are presented.

**Figure 7:**
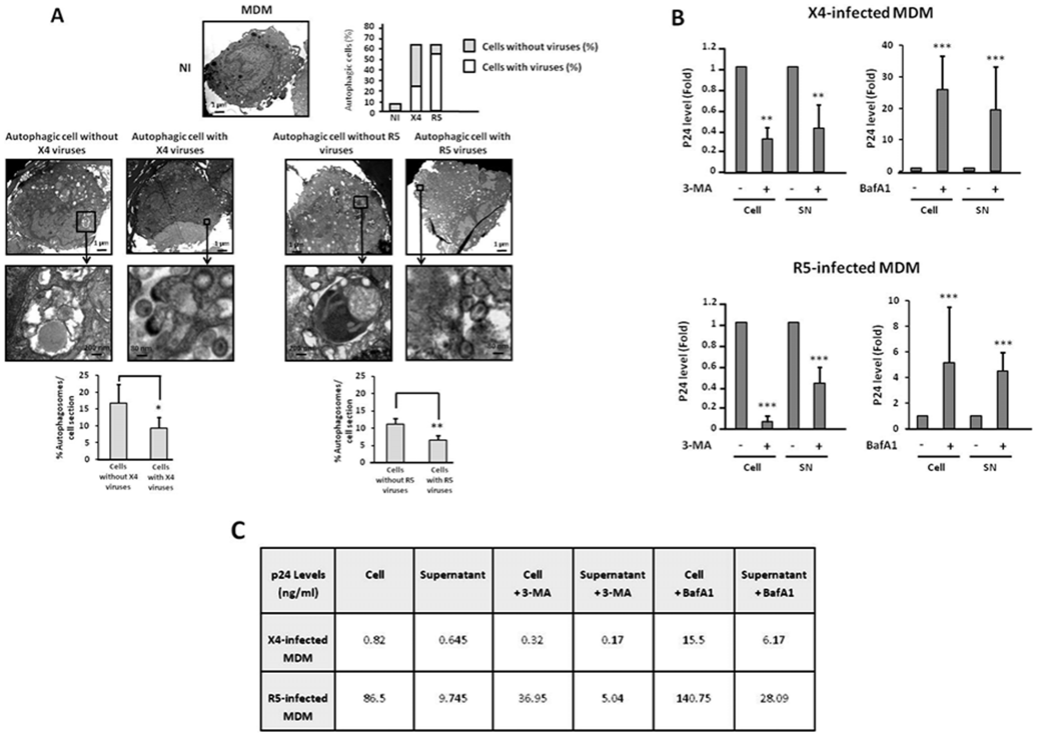
Autophagy induced in MDM after X4 or R5 infection is involved in viral replication. (A) MDM were cocultured with chronically X4 or R5-infected MOLT-4, for 3 days or 1 day, respectively, or with uninfected MOLT-4 for 3 days, and examined by TEM as described in Figure 5. The upper graph indicates the percentage of autophagic cells containing, or not, morphologically intact virions after X4 or R5 infection. Enlargement of viruses and autophagosomes are presented. (B) Ratio between the level of p24 in the supernatant or the cell lysate of X4 and R5-infected MDM in the absence of 3-MA or BafA1 and the level of p24 in in the supernatants or the cell lysates of X4 and R5-infected MDM in the presence of 10 mM 3-MA or 100 nM BafA1 for 2 days. The intracellular p24 levels were normalized with respect to the number of live cells. Data are the mean of at least 3 independent experiments. (C) Quantification of p24 (ng/mL) in the supernatant and the cell lysate of X4 and R5-infected MDM in the presence or absence of 10 mM 3-MA or 100 nM BafA1. Data are representative of at least 3 experiments.

Next, we analyzed autophagy in THP1, THP1-PMA and MDM after coculture with effector cells that produce X4 or R5 HIV-1 virions. THP1 were cocultured with HEK cells expressing infectious X4 or R5 viral particles, or with parental HEK cells. THP1-PMA and MDM were cocultured with MOLT-4 chronically infected by X4 or R5 strains. In both cases autophagosomes were present in these target cells. In contast, no autophagosomes were observed when target cells were cocultured with uninfected control cells (Figure 5, Figure 6 and Figure 7A). Interestingly, by TEM, we observed two categories of autophagic cells, independently of coreceptor use: a population of highly autophagic cells arbitrarily defined by the presence of 10 or more autophagosomes per cell section, and a population of weakly autophagic cells, with less than 10 autophagosomes per cell section. It is worth noting that the weakly autophagic cells contained very small autophagosomes. Furthermore, these cells harboured large numbers of empty vesicles. Surprisingly, viruses could be detected in the weakly autophagic cells but not in the highly autophagic cells. Interestingly, 57.4%±1.3% of MDM exposed to R5 HIV-1 were weakly autophagic, with detectable virions, and 9.6%±1.6% were highly autophagic, without detectable virions, whereas 24.8%±3.2% of MDM exposed to X4 HIV-1 were weakly autophagic, with detectable virions, and 43.3%±5.5% were highly autophagic, without detectable virions (Figure 7A). Thus, exposure of different cells from the monocyte/macrophage lineage to X4 and R5 viruses triggers autophagy, but intracellular viruses are detectable only in weakly autophagic cells.

### Autophagy induced in MDM after X4 or R5 infection plays a dual role in viral replication

To analyze the role of autophagy in viral replication after X4 or R5 infection of the MDM, we quantified the levels of p24 production in the presence of 3-MA (that blocks the initial stages of autophagy by inhibiting class III PI3 kinase) or Baf A1 (that blocks autophagosome-lysosome fusion by inhibiting the vacuolar H+ ATPase). We analyzed the levels of p24 in both the cells and the supernatants after two days of X4 or R5 infection to measure intracellular and extracellular X4 and R5 HIV-1 yields, respectively.

As previously described [23], we observed that MDM were more prone to R5 than X4 infection, since p24 was produced at a higher level in both the cell and the supernatant of MDM infected by R5 than X4 viruses (Figure 7C).

Surprisingly, when autophagy was inhibited by the addition of 3-MA, the levels of intracellular and extracellular p24 were dramatically decreased (Figure 7B and 7C), suggesting that autophagy is needed for productive X4 and R5 HIV-1 infection of MDM. In contrast, the addition of Baf A1 increased the production of X4 and R5 viruses. Importantly, BafA1 had a greater effect on X4 infection than on R5 infection (Figure 7B and 7C), suggesting that either R5 viruses are able to better control the complete autophagic process or that the ability of R5 viruses to replicate in MDM overwhelms the capacity of autophagy to control viral replication. In all cases, released virions were infectious (data not shown).

## Discussion

The major factor involved in the development of AIDS is the depletion of uninfected, bystander CD4 T cells, while the population of macrophages remains unaffected. Both of these cell types can be productively infected by HIV-1, however there are major differences in the features of this infection [24], [25]. Firstly, CD4 T cells must be activated before they can be productively infected, a process that includes cell division, while productive infection can be established in macrophages even though they are not dividing. Secondly, following HIV infection, gene expression is differentially modulated, contributing to both greater survival of macrophages and facilitation of HIV-1 replication. Thirdly, in contrast to CD4 T cells, macrophages are more prone to R5 infection than X4 infection. Fourthly, viral assembly events may also differ. Indeed, although it is clear that it occurs at the plasma membrane in CD4 T cells, in macrophages, there is still some debate. HIV-1 budding has recently been shown to take place at the plasma membrane, even if many data indicate that it buds into, and accumulates in, endocytic compartments, named multivesicular bodies (MVB) [1], [26], [27], [28], [29], [30]. Furthermore, although they are both immune cells, their functions are highly different, and thus, they have different strategies in regulating the innate and/or adaptative responses against pathogens. In particular, macrophages are able to capture and digest microbes, as well as to present antigenic peptides [27].

A lot of recent data indicates that autophagy is a fundamental antiviral mechanism [13], [14], [16]. Almost nothing is currently known, however, concerning the global role of autophagy in HIV-1 pathogenesis. We have demonstrated that autophagy is directly responsible for the destruction of uninfected CD4 T cells during HIV-1 infection by X4 strains [10] whereas autophagy is inhibited in X4 HIV-1-infected CD4 T cells and U937 cells [31]. In addition, a recent functional genomic screen in TZM-bl cells has shown that members of the two protein-conjugation pathways involved in autophagy: Atg7, Atg8, Atg12 and Atg16L2 are critical regulators of HIV infection [32].

We demonstrate here that Env from both R5 and X4 strains triggers autophagy and cell death in uninfected CD4 T cells. This result is consistent with the fact that, during acute infection, HIV-1 destroys a large number of CCR5 CD4 memory T lymphocytes in the intestinal mucosa through cytopathic and apoptotic mechanisms [33], [34], [35].

In contrast to CD4 T cells, however, uninfected macrophages do not undergo Env-mediated autophagy, whatever the coreceptor used, indicating that autophagy triggered by Env is a cell-type dependent process. This discrepancy raises unsolved questions about the mechanism that leads to Env-mediated autophagy and how it is regulated in a cell type-dependent manner. The fusogenic function of gp41 is known to be responsible for the induction of autophagy in CD4 T cells after contact with infected cells [9]. However, this event also occurs in uninfected macrophages cocultured with HIV-1-infected cells. One hypothesis to explain this discrepancy is that gp41-induced perturbations, triggered at the membrane of both macrophages and CD4 T cells, could be either different or differentially regulated. Indeed, additional macrophage-specific membrane interactions exist following Env binding to receptor/coreceptor, including annexin II, p21 and α-v-integrin [1], [36]. HIV-1 can also enter macrophages by endocytosis or after interaction with innate immune receptors such as macrophage mannose receptor [37]. Furthermore, recent data demonstrate that ceramides, that are known inducers of autophagy, play an important role in membrane protein reorganization [38], a phenomenon that is necessary for HIV-1 entry into cells.

A second hypothesis is that gp120 binding to CD4 and the coreceptor, steps that precede gp41 insertion into the target membrane, transduces signals that counteract Env-mediated autophagy in a cell-type dependent manner. We also cannot exclude a role of the secretion of chemokines and/or cytokines in regulating gp41-induced autophagy. Further investigation is needed to answer this point. Importantly, our data also demonstrate that autophagy is regulated by HIV-1. In CD4 T cells productively infected by either X4 or R5 strains, autophagy is totally inhibited. These results are in accordance with those obtained by Zhou and Spector who have demonstrated that Beclin 1 and LC3-II are decreased in X4-infected CD4 T cells [31]. Since autophagy induced by Env in the uninfected CD4 T cells leads to cell death, this result also suggests that HIV-1 is capable of counteracting Env-mediated apoptosis in infected CD4 T cells by inhibiting autophagy, thus allowing viral replication to occur. In the monocyte/macrophage cell lineage (from monocytes to differentiated macrophages), autophagy plays a more complex role. On one hand, autophagy is triggered in these cells following X4 or R5 HIV-1 infection (Figure 5, Figure 6, and Figure 7) and blockade of this process by 3-MA dramatically decreases the p24 levels, strongly suggesting that autophagy is required for viral replication in monocytes/macrophages. On the other hand, autophagy triggered in X4 and R5-infected MDM reduces HIV-1 infection. Indeed, addition of BafA1, a compound that inhibits the acidification of lysosomes and endosomes, and thus, at a later stage, lysosomal degradation [39], [40], increases HIV-1 production. This result is correlated with the observation that no virion is visible by TEM in either highly autophagic or non autophagic cells from the monocyte/macrophage cell lineage, while weakly autophagic cells contain intact virions. These results are however in contradiction with data from Zhou and Spector [31] showing autophagy to be decreased in the promonocytic cell line U937 after HIV-1 infection. Thus, HIV-1 is able to usurp the autophagic machinery in MDM, most probably to support viral replication, as already described for other viruses [16], [41], [42]. However, autophagy also plays a major role in limiting viral replication and is not totally controlled by HIV-1-infected MDM since blockade of the lysosomal degradation step increases viral production. Interestingly, both X4 and R5 strains trigger autophagy in HIV-1-infected MDM, but the percentage of weakly *versus* highly autophagic cells depends on the strain. Indeed, X4-infected MDM are mainly highly autophagic, with more than 10 autophagosomes per cell section, and thus are less infected than R5-infected MDM that are essentially weakly autophagic (less than 10 autophagosomes per cell section). This is consistent with the physiopathology of HIV-1 infection since R5 strains are more infectious than X4 strains in MDM. This suggests that the regulation of autophagy is one of the mechanisms explaining the course of infection.

One hypothesis could be that HIV-1 is still an emerging viral pathogen that is not yet totally adapted to replicate in its different host cells *i.e.* it has not “achieved” the control of autophagy in both infected and uninfected cells, essentially macrophages and CD4 T lymphocytes, respectively. We do not currently know how autophagy is controlled by the cell and/or by the virus and no data have yet brought clear responses concerning the differential susceptibilities of HIV-1 target cells to X4 and R5 HIV-1 infection. However, to the best of our knowledge, these findings represent the first example of a differential cell-type control of autophagy that governs both viral replication and the fate of uninfected cells.

In conclusion, our data strongly suggest that autophagy is responsible for HIV-1 pathogenesis, providing new insights into therapeutic strategies for the future.

## Materials and Methods

### Effector cell lines expressing Env

The HEK.X4 Env cell line, used in previous studies [8], [10], is a HEK.293 cell line stably transfected with the T-tropic HIV-1 defective pBRUΔgag construct. 2 µM Methotrexate was used as selection marker. HEK.R5 Env is a HEK.293 cell line stably transfected with the HIV-1 R5 pCMV-AD8env expression vector (kindly provided by R. Willey). The Ad8env reading frame was amplified by PCR and cloned into the Not site of pCMVb (Clontech). 250 µg/mL Zeocin was used as selection marker.

### Cell culture

The original HEK.293 cell line, as well as HEK.X4 Env and HEK.R5 Env, were cultured in DMEM supplemented with antibiotics and 10% FCS. The MOLT-4 CD4 T cell line and the chronically HIV-1-infected MOLT-4 cells were provided by J. Blanco (Barcelona, Spain). The CD4 T cell lines and THP1 cells were cultured in RPMI 1640 medium supplemented with antibiotics and 10% FCS. Monocytes were purified from the blood of healthy human donors provided by the EFS (Etablissement Français du Sang, France). Briefly, peripheral blood mononuclear cells (PBMC) were prepared by density centrifugation on Ficoll-Paque (Eurobio, Les Ulis, France). T cells were depleted by rosetting with sheep red blood cells following the protocol described in Current Protocols of Immunology. B lymphocytes and NK cells were depleted using CD19- and CD56-coated magnetic beads (Dynal, Compiègne, France), respectively. The recovered cells (referred to as monocytes hereafter) were seeded into six-well plates at a density of 0.7×10^6^/mL in complete culture medium (RPMI+10% FCS) and differentiated into macrophages with rh-M-CSF (10 ng/mL, Immunotools, Friesoythe, Germany). After 6 days of rh-M-CSF treatment, monocytes display macrophage characteristics, and are hereafter referred to as macrophages (MDM).

### Reagents and antibodies

3-MA and Baf A1 were purchased from Sigma-Aldrich. Antibodies were obtained from the following companies: anti-CXCR4 antibody (R&D Systems); anti-LC3 (Novus Biologicals); anti-CCR5 (Becton Dickinson); and anti-CD4 (Beckman Coulter).

### Flow cytometry

Cells were incubated for 1 hour at 4°C with 50 µl PBS supplemented, or not, with the appropriate Ab. After washes with PBS, bound Ab was revealed by addition of 50 µl of a 1∶100 dilution of FITC-conjugated secondary Ig. After staining for 30 minutes, cells were washed with PBS, and fluorescence intensity at 543 nm was measured on a COULTER EPICS XL Flow Cytometer (Beckman Coulter). The percentage of HIV-1-infected cells was analysed using the HIV KC57 FITC kit according to the manufacturer's instructions (Beckman Coulter).

### Western blot analysis

Cells were washed twice in PBS and lysed in buffer containing 50 mM Tris HCl (pH 8), 1% Triton X-100, 100 mM NaCl, 1 mM MgCl2, 150 µM PMSF, and complete mini protease inhibitor cocktail (Roche Diagnostics). Cell lysates were electrophoresed in 12% SDS-PAGE and blotted to PVDF membranes. After a blocking step for 1 hour at room temperature, blots were incubated for 2 hours at room temperature with the primary Ab in the blocking buffer. After 3 washes with TBS and 0.05% Tween, the blots were incubated for 1 hour at room temperature with peroxidase-coupled antiserum diluted in TBS, 5% milk, and 0.05% Tween. After further washes, the immune complexes were revealed by ECL (PerkinElmer) and autoradiographed.

### TEM analysis

Cells were treated as previously described [10] and sections were examined with a Hitachi H7100 TEM. Autophagic cells were defined as cells that had 5 or more autophagosomes (*i.e*. double membrane vacuoles containing cytosolic material). The percentage of autophagic cells in the target cell sections was quantified from at least 100 randomly chosen TEM fields by 2 investigators.

### LC3 staining

Target cells were transfected with pEGFP or pEGFP-LC3 plasmid (referred to as GFP and GFP-LC3, respectively) using the Nucleofector apparatus (amaxa biosystems) according to the manufacturer's instructions. Cells were then washed with PBS and fixed in 3.7% paraformaldehyde in PBS for 10 minutes at room temperature and examined by epifluorescence using a Leica microscope. More than 100 transfected cells were counted by 2 investigators. Only cells with at least 5 spots were counted as positive. Results are from at least 3 independent experiments.

### Detection of cell death

Cell death of the target cells was studied by trypan blue exclusion test as previously described [10].

### HIV-1 infections

CD4 T cells and THP1 were cocultured with plated HEK.293 cells previously transfected with 5 µg of X4 NL4-3 or the R5 NL4-Ad8 DNA constructs using Jet Pei (Ozyme) according to the manufacturer's instructions. THP1-PMA and MDM were cocultured with MOLT-4 cells chronically infected by X4 or R5-strain.

HIV-1 infections were also performed with normalized amounts of supernatants of X4 or R5 HIV-1-transfected cells. 10^6^ MDMs were infected with 500 µL of a viral solution containing 300 ng/mL p24 for 2 hours at 37°C in presence or absence of 10 mM 3-MA or 100 nM BafA1. Cells were then washed 6 times in complete medium and cultured for 2 days in 2 mL of complete medium containing M-CSF with or without 3-MA or BafA1. Infection was followed by measuring HIV-1 gag p24 in both the supernatants and cell lysates of the infected cells using a p24 antigen capture ELISA (Beckman Coulter) [43]. The intracellular p24 levels were normalized with respect to the number of living cells.

### Statistics

Analysis of the variance of the results was performed after arc sine transformation of the data when percentages were compared [44]. Differences were considered significant at *P<0.05, **P<0.01, and ***P<0.001.
